# Atomoxetine treatment for nicotine withdrawal: a pilot double-blind, placebo-controlled, fixed-dose study in adult smokers

**DOI:** 10.1186/1744-859X-11-6

**Published:** 2012-03-09

**Authors:** Peter H Silverstone, Rana Dadashova

**Affiliations:** 1 Department of Psychiatry, University of Alberta, Edmonton, Alberta, Canada

**Keywords:** adults, atomoxetine, clinical trial, double-blind, nicotine, placebo-controlled, smoking

## Abstract

**Background:**

Many effective treatments for nicotine addiction inhibit noradrenaline reuptake. Three recent studies have suggested that another noradrenaline reuptake inhibitor, atomoxetine, may reduce smoking behaviors.

**Methods:**

The present double-blind, placebo-controlled, fixed-dose study was carried out over 21 days during which administration of 40 mg atomoxetine was compared to placebo in 17 individuals. Of these, nine were randomized to atomoxetine and eight to placebo. Baseline and weekly measurements were made using the Cigarette Dependence Scale (CDS), Cigarette Withdrawal Scale (CWS), Questionnaire of Smoking Urges (QSU), reported number of cigarettes smoked, and salivary cotinine levels.

**Results:**

The study results showed that all those on placebo completed the study. In marked contrast, of the nine individuals who started on atomoxetine, five dropped out due to side effects. In a completer analysis there were statistically significant differences at 14 and 21 days in several measures between the atomoxetine and placebo groups, including CDS, CWS, QSU, number of cigarettes smoked (decreasing to less than two per day in the treatment group who completed the study), and a trend towards lower mean salivary cotinine levels. However, these differences were not seen in a last observation carried forward (LOCF) analysis.

**Conclusions:**

In summary, this is the first study to examine the use of atomoxetine in non-psychiatric adult smokers for a period of more than 7 days, and the findings suggest that atomoxetine might be a useful treatment for nicotine addiction. However, the dose used in the current study was too high to be tolerated by many adults, and a dose-finding study is required to determine the most appropriate dose for future studies of this potential treatment for smoking cessation.

## Introduction

Smoking is recognized as a global concern. Nicotine addiction affects not only tobacco users, but also their families and society in general. Impacting both mental and physical health, smoking addiction generates high costs associated with multidimensional measures, significantly affecting economy of countries worldwide [1]. It has been predicted that annual worldwide smoking-related deaths will reach roughly 3 million people per year in industrialized countries and 7 million per year in developing countries by 2030 [2].

For several decades researchers have been attempting to find effective therapies for smoking cessation. Several studies have demonstrated that alterations in noradrenaline (NA) neurotransmission are a contributor to the process of nicotine dependence [3-5], smoking maintenance [6], relapse [7], and withdrawal [3].

The neurobiology of addiction to multiple different drugs has been explained primarily through the mechanisms underlying the reinforcing and rewarding properties of the specific addictive substances. The relationship between the noradrenergic system and rewards mechanisms have been reported by a number of authors who have pointed out that the major noradrenergic nucleus, the locus ceruleus, sends its projections to ventral tegmental and nucleus accumbens, thus influencing these reward and reinforcing pathways [8-10]. Moreover, the release of dopamine in the nucleus accumbens induced by all addictive substances is considered to be a common mechanism in the development and maintenance of the addiction; however, it has also been found that the firing of midbrain dopamine cells is modulated by noradrenergic neurons from the locus ceruleus [11]. This discovery, along with other research, led Weinshenker and Schroeder to propose the role of noradrenaline pathways in stimulant additions [12].

Although the exact role of noradrenaline in nicotine dependence is still uncertain, it has been demonstrated that drugs which are efficacious at inhibiting NA reuptake are also frequently clinically effective in treating nicotine dependence, and are recognized as first line agents in treatment [13,14]. Nonetheless, the efficacy of current pharmaceutical aids remains wanting, and there is currently a great need for other agents.

Towards this goal it has been suggested that another noradrenaline reuptake inhibitor, atomoxetine, which is used in the treatment of attention deficit disorder [15], may also be useful [16]. Atomoxetine's clinical benefits are believed to be due to noradrenergic augmentation in the prefrontal cortex [17-19]. Atomoxetine also possesses a low affinity for multiple other neurotransmitters including serotonin, dopamine, choline, γ-aminobutyric acid (GABA), adenosine transporters, and ion channels [15,20-22]. Thus, actions of atomoxetine that increase levels of other neurotransmitters may also have an indirect effect mediated via increased noradrenaline release [23,24]. It is therefore conceivable that mechanism of action could have potential additional benefits in the treatment of nicotine addiction. Furthermore, its inability to raise the concentration of dopamine in the prefrontal cortex is probably linked to its low abuse potential, a finding that has been supported in clinical trials [15,23-25].

There have been three previous studies to date which have examined the possible effectiveness of atomoxetine for smoking cessation, all in patients who have a psychiatric diagnosis in addition to nicotine addiction, one in non-psychiatric patients over 7 days, one in patients with attention deficit hyperactivity disorder (ADHD), and one in patients with schizophrenia [16,26,27]. All give tentative support to the possibility that atomoxetine can reduce consumption and/or smoking behaviors. However, to date there have been no studies examining the possible efficacy of atomoxetine in smoking cessation in adult individuals who only have a nicotine addition over more than 7 days. Given the need for additional treatments the present study was designed to determine if such a treatment warrants further investigation.

## Methods

The objectives of the study were to determine if more subjects in the atomoxetine treatment group had less severe withdrawal symptoms, and smoked fewer cigarettes, during a 21-day study period than those who received placebo. The study was approved by the Health Ethics Committee of the University of Alberta.

The length of time that subjects were studied (21 days) was based upon findings showing that most individuals who try and stop smoking relapse during this period [28]. While it is clear that larger phase III smoking cessation studies have examined patients for 12 weeks for drugs such as varenicline [29-31], other pilot studies of potential treatments have suggested a window of 3 to 4 weeks is sufficient at the early stages of development [32].

### Study population

The study population was defined as healthy volunteers who wanted to quit smoking and met the *Diagnostic and Statistical Manual of Mental Disorders*, fourth edition (DSM-IV) criteria for nicotine dependence. They were screened to ensure they met inclusion criteria and did not have any criteria for exclusion (see below).

### Inclusion criteria

The following inclusion criteria were used in the study: (1) patients who want to quit smoking and who signed an informed consent form approved by the ethics committee; (2) diagnosis of nicotine dependence according to DSM-IV criteria [33]; (3) patients must be smoking between 10 to 25 cigarettes per day, and have done so for at least the previous 12 months (while the choice of the degree of nicotine dependence in this study was somewhat arbitrary, this is consistent with the average number of cigarettes smoked per day in Canada currently being approximately 13; this is also consistent with most smokers being in the 'light' range, since the US National Institute on Drug Abuse classifies smoking levels as light (less than 15 cigarettes per day), moderate (15 to 24 cigarettes per day) and heavy (25 and more cigarettes per day)); (4) age between 21 to 60 years old (this age range was chosen since Canadian statistical data for last decade showed that the majority of current smokers belonged to the age groups that fall between 20 and 55 years of age [34]). Note that individuals aged between 18 to 21 were specifically excluded, as we were concerned that they may not have an established smoking pattern and therefore withdrawal symptoms may be less likely to have manifested.

### Exclusion criteria

The following exclusion criteria were used in the study: (1) any current Axis I or Axis II psychiatric disorder (this selection criterion was used to exclude other major psychiatric conditions in the study group so as to avoid potential issues with applicability of the study: to ensure this a semistandardized interview, the Mini International Neuropsychiatric Interview (MINI) compatible with DSM-IV diagnostic criteria [35] was performed on all potential subjects); (2) history of intolerability, hypersensitivity or allergy to atomoxetine, or use of atomoxetine within 30 days of screening; (3) presence of disorders that could conceivable be exacerbated by atomoxetine (specifically, narrow angle closure glaucoma, urinary outflow obstruction, hypertension, and neurological disorders, particularly tics and Tourette's syndrome, or a history of epilepsy or seizures); (4) use of concomitant medication that could potentially interact with atomoxetine including monoamine oxidase inhibitors (MAOI), antihypertensive medication, or any concomitant medication that was a cytochrome 2D6 inhibitor (CYP2D6), since atomoxetine's elimination involves the CYP2D6 system; (5) use of any recreational or illegal drugs in the 3 months prior to the study, of individuals who met criteria for alcohol dependence or alcohol abuse in the 3 months prior to the study; (6) presence of any suicidal ideations during screening testing.

### Screening and baseline

To ensure that patients met inclusion criteria the following baseline information was collected and a physical examination was also completed: demographic data: gender, age, educational level, income, and occupation, marital status, and ethnicity; smoking history and determine that the individuals met diagnostic criteria for nicotine dependence according to DSM-IV classification; psychiatric history including other substance use/abuse; the Mini International Neuropsychiatric Interview; medical history; and concomitant medications.

### Outcome measurements

The following five outcome measurements were employed to determine baseline ratings for subjects, and how they subsequently changed: (1) severity of nicotine dependence utilizing the Cigarette Dependence Scale (CDS) [36,37]; (2) withdrawal symptoms utilizing the Cigarette Withdrawal Scale (CWS) [38,39]; (3) smoking urges and craving utilizing the Questionnaire for Smoking Urges (QSU) [40-42]; (4) Self-monitoring diary of smoking, which was completed daily by subjects (this reflected the subject's assessment of how much they were smoking daily); (5) salivary cotinine measurements, as recommended in previous studies [43-46]. Cotinine is the major metabolite of nicotine and accounts for approximately 75% of all byproducts of nicotine metabolism [47,48]. Cotinine can be detected in diverse body fluids such as urine, serum [49], cervical mucus [50], semen [51], saliva [52-54], and amniotic fluid [55].

In addition to the outcome measurements participants underwent a repeat physical examination that included measurement of vital signs (blood pressure, pulse, temperature) and weight.

Both treatment and placebo groups received the following advice and information about stopping smoking, and these were repeated at each visit: (1) advised to discuss quitting attempt with family members; (2) to anticipate challenges during quitting attempt; (3) likely withdrawal symptoms, and triggers for these; (4) to remove tobacco products from their home; (5) if there were others in their home who smoked, to try and get them to also stop; (6) to ask other smokers in the same household not to smoke in their presence and/or only smoke outside of the house; (7) to stop alcohol use (exclusion criteria in our research and trigger); (8) discussed health risks and rewards that quitting provides; (9) provided with information about programs and resources for smokers within the city.

### Randomization and details of measurements at each visit

In order to avoid selection bias, the assignment of subjects to the two study groups was randomized by a computer-generated code and this study was carried out in a double-blind manner. Participants were randomized and assigned to either atomoxetine or placebo-treatment arm during the baseline visit. Coding of study subjects (study subject number) was performed by assigning a unique alphanumeric sequential code to each participant.

After signing an informed consent form, the subjects underwent a screening interview that included a screening questionnaire consisting of demographic data, smoking history, medical history, psychiatric history, and social history of the patient, a screening questionnaire with study inclusion and exclusion criteria, the structured clinical interview for Diagnostic and Statistical Manual (DSM-IV) to confirm that the subject met appropriate diagnostic criteria of DSM-IV for nicotine dependence, and the MINI to exclude other psychiatric disorders.

After completion of the interview subjects had a physical examination that included the following measurements: weight (kg), blood pressure (mmHg), pulse (bpm) and temperature.

Subjects also underwent a cotinine saliva test to determine their smoking status and baseline levels. Those volunteers, whose screening interview and cotinine saliva test showed that they were eligible for trial participation, were entered into the study.

The baseline visit occurred within 7 days of the screening visit. Complete information about smoking history along with detailed information on medical, psychiatric, and social history of each participant were collected during this visit. This was to complete any missing information from the screening visit. To ensure the greatest level of accuracy for the outcome measurements in our study we combined self-reported questionnaires and a self-monitoring diary with objective measurement of a biological marker for smoking, namely the cotinine saliva test, which was carried out at every visit as were all the outcome measurements.

At the baseline visit (day 1, week 0) participants underwent randomization and were assigned to either atomoxetine or placebo-treated group. Both at baseline, and each subsequent visit, the study drug was dispensed as a 7 ± 2 day supply, with a total of nine capsules in each container to allow for delayed visits.

Subjects were instructed to return for follow-up after a 1-week period (± 2 days) and bring the container and the rest of the pills (if any). Participants were also instructed about the administered daily dose of the atomoxetine, with each subject receiving 40 mg once a day.

All subjects were provided with information and brochures about support services and self-help groups for individuals who are in the process of quitting smoking.

Subjects were also instructed that follow up visit will be held every 7 days (± 2 days) during the 21-day treatment period. Thus, each subject had three additional follow-up visits (day 7, week 1; day 14, week 2; day 21, week 3). Participants were advised to report all side effects to study medication and reminded that they were free to drop out of the study at any time if they wished. Participants underwent a repeat physical examination that included measurement of vital signs (blood pressure, pulse, temperature) and weight. At each visit the cotinine test on a salivary sample was repeated to monitor compliance and to verify self-reporting of smoking. It should be noted that the test used (NicAlert) is not a linear scale as a score of 1 represents 1 to 10 ng/ml while a score of 5 represents 500 to 2,000 ng/ml. This is therefore an indication, not a direct measurement, of serum cotinine levels. Therefore, statistical comparisons of changes with this test are only useful to give an indication of a reduction (or lack of this), rather than give definitive changes in serum cotinine levels.

At each visit adverse events were collected as was the self-monitoring diary, and a new one was provided for the next week. Participants were instructed to fill out the diary every day and return it during the next follow-up visit. Compliance with medication was also calculated. In situations where pills were returned or treatment interruption occurred, volunteers were interviewed and the reasons such as relapse or adverse event were recorded. Concomitant medications were also recorded.

### Statistical analysis

In this study we used a t test to compare the results between the group, using both the completer data and the results on a last observation carried forward (LOCF) basis to compare the mean scores for each group at each visit. Results were considered statistically significant at the *P *< 0.05 level.

We utilized a two-sample t test to compare results between the groups at each time point. This was performed both for the group of individuals who completed each visit ('treatment group') as well as for those who stopped the study early and whose missing data was completed using an LOCF analysis ('LOCF treatment').

These are shown for each of the five key outcome measures, the CDS, CWS, QSU, the number of cigarettes smoked per week (NCSW), and cotinine salivary levels.

### Sample size calculation

In order to determine the differences between treatment and placebo groups, the sample size was calculated by a power analysis. This was based on the reduction in self-reported smoking urges as measured by the QSU in a previous study of the effects of atomoxetine in smoking cessation [16]. In this study the baseline QSU score was 72 with a standard deviation of 19, while following treatment with atomoxetine it was 82 with a standard deviation of 17. Assuming that the study should be powered sufficiently to detect a similar change in magnitude, a sample size calculation was made using these values and an α error level of 5% (corresponding to a 95% confidence interval) and a β error level of 10% (the probability of incorrectly failing to reject the null hypothesis that there is no difference in the average values). Using this information the number of subjects required to reach statistical significance was calculated as 56. To account for dropouts, a total of 60 subjects in each arm were felt to be required. Thus, it was the intention to recruit a total of 120 subjects, 60 in each arm.

## Results

### Recruitment and demography

A total of 34 potential subjects attended for a screening visit. However, 14 did not meet inclusion and exclusion criteria. Thus, a total of 20 people entered the study. Of these 20, 3 individuals attended the baseline visit, but did not take any study medication and were therefore not included in the study. The study population, therefore, consists of 17 subjects who were randomized to receive double-blind medication. Of these, a total of five dropped out during the study, all due to side effects. In all, 12 subjects completed the study.

The demographic characteristics of the study participants are summarized in Table 1. There were no statistically significant differences between the groups.

**Table 1:** Demographic characteristics of study participants

Characteristics	Atomoxetine group (N = 9; 52.9%)	Placebo group (N = 8; 47.1%)	*P *value
Age, years:			
Mean (SD)	40.9 (11.9)	43 (9.9)	0.699^a^
Range	25 to 57	31 to 58	
Gender, n (%):			
Male	5 (55.6%)	3 (37.5%)	0.637^b^
Female	4 (44.5%)	5 (62.5%)	
Ethnicity, n (%):			
White	8 (88.9%)	7 (87.5%)	1.000
Other^c^	1(11.1%)	1 (12.5%)	
Education, n (%):			
High school	2 (22.2%)	0 (0%)	0.471
College/university	7 (77.8%)	8 (100%)	
Occupation, n (%):			
Student/unemployed	3 (33.3%)	1 (12.5%)	0.576
Employed	6 (66.7%)	7 (87.5%)	
Income, n (%):			
Low/moderate	7 (77.8)	7 (87.5%)	1.000
High	2 (22.2%)	1 (12.5%)	
Marital status, n (%):			
Married/common law	5 (55.6%)	1 (12.5%)	0.131
Single/divorced	4 (44.5%)	7 (87.5%)	

^a^The t test was used to compare the means ages of both groups.

^b^The Fisher's exact test for used to compare the proportions.

^c^The other subgroup incorporates the following ethnicities: one Asian in the atomoxetine group and one Métis individual in the placebo group.

### Clinical characteristics of study participants

There were no statistically significant differences between clinical characteristics and the family history of participants in both treatment groups. Thus, there were no statistically significant differences between the two groups in terms of history of mental disorders; comorbid general medical conditions; health concerns/problems related to smoking; alcohol use or abuse; use of medications for the treatment of medical conditions throughout the study; family history of mental health conditions other than nicotine dependence; or family history of smoking.

### Smoking-related history for all participants

All 17 participants met criteria for nicotine dependence according to DSM-IV classification. In terms of specifics, all of them had nicotine dependence with physiological dependence. A summary of their current and past smoking behavior is shown in Table 2.

**Table 2:** Smoking data for all study participants

Characteristics	Atomoxetine arm (N = 9)	Placebo arm (N = 8)	*P *value
Length of every day smoking, years:			
Mean (SD)	20.9 (11.3)	23.1 (8.4)	0.655
Range	5 to 40	11 to 36	
Number of cigarettes per day, n (%):			
Mean (SD)	19.1 (5.5)	19.1 (5.0)	0.979
Range	11 to 27.5	12 to 27	
Age of smoking initiation, years:			0.984
Mean (SD)	16.3 (2.8)	16.4 (5.8)	
Range	13 to 21	11 to 29	
Number of quitting attempts:			
Mean (SD)	4.7 (3.2)	5.9 (5.8)	0.596
Range	2 to 10	2 to 20	
Median length of the recent attempt to quit smoking, days:			
Median	3	13	0.409
Range	2 to 3,600	3 to 180	
Percentile 25	2	7	
Percentile 50	3	13.8	
Percentile 75	510	120	
Median of average length of quitting attempts, days:			
Median	60	19.3	0.630
Range	2 to 7,200	1 to 360	
Percentile 25	2.25	11.4	
Percentile 50	60	19.3	
Percentile 75	2,205	78	
Previous use of smoking cessation medications, n (%):	8 (88.9%)	7 (87.5%)	1.000
Help of smoking cessation medication, n (%):	6 (66.7%)^a^	7 (87.5%)^a^	0.467

^a^One smoker from atomoxetine treatment group and one smoker from placebo group did not use any medication for smoking cessation.

Among all study participants, only one individual in the atomoxetine group and three individuals in the placebo group reported the development of respiratory problems related to smoking. One individual in the placebo group was diagnosed with chronic obstructive pulmonary disease (COPD).

There were no statistically significant differences between the two treatment groups (Fisher's exact test and non-parametric Mann-Whitney tests).

### CDS scores

**Figure 1:**
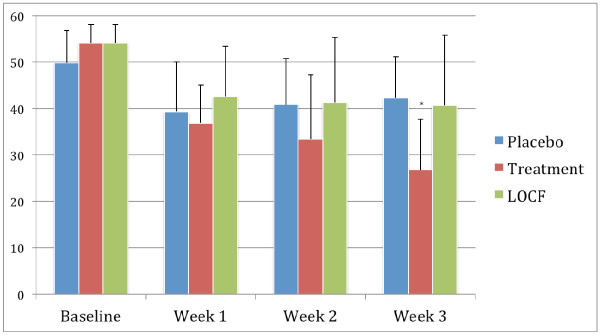
**Mean scores on the Cigarette Dependence Scale (CDS)**. The figure shows the changes in the mean scores on the CDS over the 3-week study period in the group receiving placebo (placebo group), those receiving atomoxetine who completed the study (atomoxetine group), and those who received atomoxetine but dropped out during the study (last observation carried forward (LOCF) atomoxetine group). A two-sample t test demonstrated statistically significant difference (*P *< 0.05) between the mean scores in the atomoxetine group compared to the placebo groups at 3 weeks (*).

The changes over time for the CDS scale are shown in Figure 1. The results showed that there was a statistically significant difference between the atomoxetine treatment arm compared to those in the placebo group at the end of the study (mean score of 26.8 vs 42.3 respectively, *P *< 0.05). However, this difference was not statistically significant in an LOCF analysis.

### CWS scores

**Figure 2:**
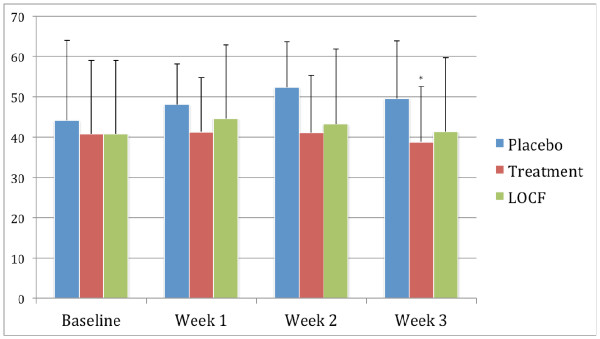
**Mean scores on the Cigarette Withdrawal Scale (CWS)**. The figure shows the changes in the mean scores on the CWS over the 3-week study period in the group receiving placebo (placebo group), those receiving atomoxetine who completed the study (atomoxetine group), and those who received atomoxetine but dropped out during the study (last observation carried forward (LOCF) atomoxetine group). A two-sample t test demonstrated statistically significant difference (*P *< 0.05) between the atomoxetine group and placebo groups at 3 weeks (*).

The changes over time for the CWS scale are shown in Figure 2. In the placebo group there was an increase in the severity of withdrawal symptoms of 4.5 during the study period, with a peak increase of 8.2 at week 2. In contrast, in the atomoxetine group there was a decrease of 2.0 during the study, and at week 2 the peak increase was only 0.4. These differences were statistically significant (*P *< 0.05) in the completer group. However, when the LOCF measurements were made, the differences between the groups were no longer statistically significant. These results suggest that those study participants who completed the study and received atomoxetine may have experience a reduction in their nicotine withdrawal symptoms compared to those who received placebo, but the data is by no means clear in this regard.

### QSU score

**Figure 3:**
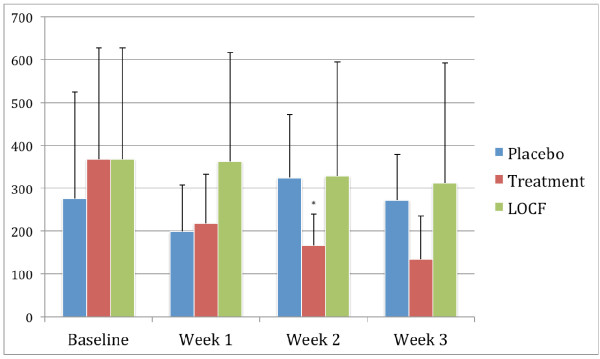
**Mean scores on the Questionnaire of Smoking Urges (QSU) scale**. The figure shows the changes in the mean scores on the QSU over the 3-week study period in the group receiving placebo (placebo group), those receiving atomoxetine who completed the study (atomoxetine group), and those who received atomoxetine but dropped out during the study (last observation carried forward (LOCF) atomoxetine group). A two-sample t test demonstrated statistically significant difference (*P *< 0.05) between the atomoxetine group and placebo groups at 2 weeks (*), but this just failed to reach statistical significance at 3 weeks.

The changes over time for the QSU scale are shown in Figure 3. It can be seen that at week 2 there is a reduction in the QSU scores in the treatment group (from a baseline measurement of 377 to 134), and this was accompanied by a statistically significant difference between the atomoxetine treatment arm and placebo treatment arm at week 2 (*P *= 0.049). However, the difference narrowed at week 3 and just failed to reach statistical significance at the final visit (*P *= 0.058). Overall, the QSU scores did not change in the placebo group, while there was a marked reduction in those individuals received atomoxetine and who completed the study.

In contrast, there were no statistically significant differences between the placebo group and the LOCF treatment group at any time point, and at week 1 the LOCF groups scores were in fact higher, although this was not statistically significant (*P *= 0.115).

### Number of cigarettes smoked per day

**Figure 4:**
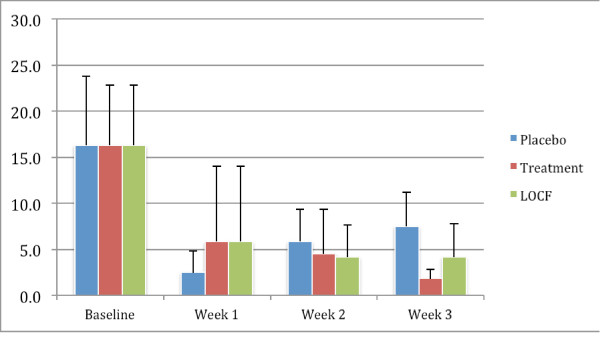
**Mean number of cigarettes smoked in period between visits**. The figure shows the changes in the mean number of cigarettes smoked each day at baseline, and then during the 6 days between each visit. These are shown for the group receiving placebo (placebo group), those receiving atomoxetine who completed the study (atomoxetine group), and those who received atomoxetine but dropped out during the study (last observation carried forward (LOCF) atomoxetine group). It can be seen that there was a marked drop in all groups. A two-sample t test demonstrated statistically significant difference (*P *< 0.05) between the atomoxetine group and placebo groups during this last measurement period (*).

Changes in the self-reported number of cigarettes smoked are shown in Figure 4. The mean number of cigarettes smoked per day by patients before they started was 16.3 in the placebo group and 16.4 in the treatment group. This decreased considerably in both groups during the first week, but whereas it started to increase back up towards baseline in the placebo group, it continued to decrease in the treatment group. This data would be consistent with individuals on placebo decreasing their smoking during the first week of their participation, but their smoking rates then increased back to 7.4 per day as the study progressed. In contrast, in the treatment group who remained on atomoxetine the number of cigarettes smoked daily continued to decrease throughout the study with them mean number of cigarettes smoked in the treatment group being 1.8 at the end of the study, as reported in their daily diaries.

This difference at the end of the study was statistically significant (*P *= 0.015). Nonetheless, when the last observation carried forward (LOCF) analysis was considered, there were no statistically significant differences between the atomoxetine group and the placebo group.

It is also important to note that none of the participants stopped smoking completely.

### Salivary cotinine

When comparing the mean levels of salivary cotinine in the two groups at week 3 there was a trend towards a statistically significant difference between those who had taken atomoxetine throughout the study and the placebo group with a mean decrease in scores from 4.1 to 2.8 compared to 4.5 to 3.8 (*P *= 0.069). However, it should be noted that the reduction from 4.1 to 2.8 in the treatment group in fact represents a significant decrease in cotinine levels from a level of approximately 200 ng/ml (represented by a score of 4 with this test) to approximately 80 ng/ml (represented by a score of 2.8).

Thus, the direction of changes is consistent with the reported reduction in the number of cigarettes smoked in the atomoxetine treated group compared to the placebo group. Nonetheless, it should be noted that when using LOCF there were no statistically significant differences between the atomoxetine treatment group and the placebo group.

### Other measurements

There was a statistically significant increase in both systolic and diastolic blood pressure in the atomoxetine group compared to the placebo group (Table 3). There were no statistically significant differences between the groups for heart rate, respiratory rate, temperature, or body weight (Table 3).

**Table 3:** Changes for study participants during the study

Characteristics	Atomoxetine arm (N = 9)	Placebo arm (N = 8)	*P *value (if significant)
Systolic blood pressure, mmHg:			
Baseline	116.4	107.5	
At end of study (LOCF)	120.6	102.8	0.007
Diastolic blood pressure, mmHg:			
Baseline	72.8	68.1	
At end of study (LOCF)	76.7	68.9	0.009
Heart rate, beats/min:			
Baseline	66.8	70.0	
At end of study (LOCF)	69.6	72.1	
Respiratory rate, breaths/min:			
Baseline	14.7	14.8	
At end of study (LOCF)	16.2	15.3	
Body temperature smoking, °C:			
Baseline	36.4	36.1	
At end of study (LOCF)	36.5	36.3	
Mean weight, kg:			
Baseline	80.9	67.9	
At end of study (LOCF)	81.4	68.5	

LOCF = last observation carried forward.

Analysis showed that individuals who dropped out from the study were not significantly different from those who completed the study in terms of comorbid general medical conditions (*P *= 0.728), use of medications for the regular treatment of medical problems (*P *= 0.169), use of alcohol (*P *= 0.536), use of other addictive substances (*P *= 0.536), or in terms of history of mental health disorders, either personal (*P *= 0.362) or family (*P *= 0.610).

### Adverse events reported by participants in the study

Several participants discontinued their participation during the study due to adverse events (Table 4). When the data was analyzed it became apparent that all discontinuations occurred in those receiving atomoxetine, due to side effects experienced by individuals. Three participants dropped out during the first week of taking the drug, one individual dropped out during the second week, and one more participant discontinued his participation during the third week of the study. Thus, more than 50% of those started on atomoxetine were not able to tolerate it at the dose used, which is the recommended starting dose for adults who have ADHD.

**Table 4:** Adverse events recorded during the study among participants

Adverse events	Total	Atomoxetine (n = 9)	Placebo (n = 8)
Insomnia	7	5 (55.6%)	2 (25.0%)
Anxiety/nervousness	2	3 (33.3%)	0 (0%)
Dry mouth	3	3 (33.3%)	0 (0%)
Dizziness	5	2 (22.2%)	3 (37.5%)
Fatigue	4	2 (22.2%)	2 (25.0%)
Nausea	4	2 (22.2%)	2 (25.0%)
Decreased concentration	4	2 (22.2%)	2 (25.0%)
Headache	4	2 (22.2%)	2 (25.0%)
Diaphoresis	3	2 (22.2%)	1 (12.5%)
Urinary retention	2	2 (22.2%)	0 (0%)
Increased energy, feeling 'high'	2	2 (22.2%)	0 (0%)
Paresthesia (tingling sensation)	2	2 (22.2%)	0 (0%)
Restless	2	2 (22.2%)	0 (0%)
Irritability	2	2 (22.2%)	0 (0%)
Sadness/low mood	2	1 (11.1%)	1 (12.5%)
Common cold	2	1 (11.1%)	1 (12.5%)
Diarrhea	1	1 (11.1%)	0 (0%)
Constipation	1	1 (11.1%)	0 (0%)
Sexual dysfunction	1	1 (11.1%)	0 (0%)
Chills	1	1 (11.1%)	0 (0%)
Tense	1	1 (11.1%)	0 (0%)
Feeling that time goes fast/speedy	1	1 (11.1%)	0 (0%)
Decreased appetite	1	1 (11.1%)	0 (0%)
Recklessness	1	1 (11.1%)	0 (0%)
Stomach pain	1	1 (11.1%)	0 (0%)
Fall	2	0 (0%)	2 (25.0%)
Sinusitis	1	0 (0%)	1 (12.5%)
Bladder infection	1	0 (0%)	1 (12.5%)
Heart palpitation	1	0 (0%)	1 (12.5%)
Other (assault, car accident)	4	2 (22.2%)	0 (0%)

In total there were 65 adverse events reported, although none of them were serious adverse events. The most frequent adverse event was insomnia, followed by dizziness, fatigue, nausea, decreased concentration, and headache.

## Discussion

This study was a randomized, placebo group of the possible use of atomoxetine to help nicotine addiction, something that represents a major health problem to many societies. It is also the first study to examine the possible efficacy of atomoxetine in adults with no other psychiatric diagnoses for up to 3 weeks.

Despite the significant limitations outlined, the results were of interest, being the first double-blind, placebo-controlled, study to examine the effects on atomoxetine in individuals who only have a diagnosis of nicotine addiction. Furthermore, there were several indications that in those individuals who could tolerate the side effects of atomoxetine, there may be clinically relevant benefits compared to placebo.

Thus, in those subjects who stayed on atomoxetine for 21 days there were several benefits that, taken together, may suggest this drug is potentially effective. Thus, these individuals had a reduction on the CDS (from a mean of 54 to 27); had a reduction on the CWS (2.0) compared to an increase in the placebo group (5.5); had a marked reduction on the QSU (from 368 to 134) while the placebo group had a very minor reduction (from 276 to 272); had a marked reduction in the number of cigarettes smoked during the study (from 43 to 11) while the numbers smoked in the placebo group increased; consistent with this large drop in the reported number of cigarettes smoked the atomoxetine group had a reduction in cotinine levels of 1.3 while the placebo group had a decrease of only 0.7.

There have been three previous studies to date that have examined the possible effectiveness of atomoxetine for smoking cessation, all in patients who have other psychiatric diagnoses. Thus, in a 2-day blinded crossover study in 15 adults with ADHD who smoke, it was found that their usual ADHD medication reduced withdrawal symptoms overnight [26], and of this group 4 patients were on atomoxetine (25 to 40 mg/day). In a 7-day double-blind crossover study in 50 individuals without a psychiatric disorder it was found that atomoxetine treatment (25 mg/day for 2 days then 40 mg/day for 2 days then 1.2 mg/kg) led to a reduction in subjective withdrawal symptoms and in smoking urges among smokers treated with atomoxetine compared to placebo [16]. In a small double-blind study in patients with schizophrenia who smoked, either placebo (n = 5), or atomoxetine at 40 mg/day (n = 4), or atomoxetine at 80 mg/day (n = 3) were administered for 14 days [27]. In these patients no statistically significant changes in smoking behaviors, as measured by smoking consumption or carbon monoxide (CO) levels were observed, although a 22% decrease in number of cigarettes per day and a 35% decrease in CO levels between baseline and day 8 in subjects taking the 80 mg/day dose were noted.

The findings from the present study are consistent with these initial findings. However, there were significant methodological differences between the studies, particularly when comparing to the most similar study, that by Ray and colleagues [16]. This latter study used the Fagerstrom Test for Nicotine Dependence as their primary outcome measure, and additionally a different measure of withdrawal was also used, the withdrawal symptom checklist (WSC) [16]. Another major methodological difference was that in the current study participants were asked to stop smoking at baseline, and then their withdrawal symptoms and smoking urges were monitored over the following 3 weeks. In contrast, Ray and colleagues did not ask the subjects to stop smoking, but observed changes in withdrawal symptoms while allowing them to continue to smoke [16]. This approach may, in part, explain the differences in the QSU score. Further differences were that in the present study a ten-item version of QSU scale was used, based upon an analysis carried out by Toll and colleagues [41]. Also, after obtaining permission from the originator of the scale, Dr. Tiffany, we received his original scoring version that was based on a 0 to 100 rating scale. In contrast, Ray and colleagues examined the QSU using a 32-item scale with response scale ratings from 1 to 7. The problems with this approach were illustrated by Toll and colleagues, who concluded that there was no consistency in term of the scoring and type of this questionnaire [36]. These differences illustrate the difficulty of directly comparing results across studies that have such different methodologies. They also illustrate the need for a more standardized approach to measuring the effectiveness, or otherwise, of atomoxetine on smoking behaviors.

In terms of adverse events, several large studies [56-62] have reported the following as the most commonly reported adverse events associated with the use of atomoxetine in adults as: dry mouth (16% to 55%), decreased appetite (12% to 50%), insomnia (17% to 35%), nervousness (35%), constipation (7% to 20%), erectile dysfunction (5% to 11%), nausea (12% to 40%), dizziness (6% to 15%), decreased libido (7%), sweating (5% to 20%), fatigue (16% to 25%), increased heart rate (17%), hypertension (10%), hot flashes (10%), depression (10%), and urinary problems (6 to 10%). The adverse events experienced by participants in the current study are compatible with these findings.

Nevertheless, the study still has several limitations and potential biases that will need to be discussed when the results are considered.

First of all, the number of participants was small, although appropriate for a pilot study. Nonetheless, the small size of the sample makes it difficult to generalize the results more widely. The sample size analysis indicated that up to 120 individuals were required for a fully powered study.

The second major limitation of the study is linked to this first point, and that is that the statistical findings were far from robust. Because of the small numbers it is difficult to be confident that even where statistically significant findings were found that they have clinical relevance. Furthermore, there were different findings when completers only were considered as opposed to the last observation carried forward analysis. This finding emphasizes that the results are preliminary, and should be taken as possibly indication of utility only. They should not be taken as support for using this drug in individuals until more robust data is available.

A third limitation was the large dropout rate among the atomoxetine treatment group. The recommended starting dose for atomoxetine in adults with ADHD is 40 mg. This was the dose used in the present study. However, less than 50% of those who started on this dose were able to tolerate it for 3 weeks. If this study was to be repeated it would be strongly recommended that a lower dose was used, possibly with a slow dose titration over 1 or 2 weeks. The large number of side effects experienced by those who took part in the study is a testament to how significantly otherwise healthy individuals were affected. Of interest is the fact that this dose is recommended for all teens and adults over 70 kg, but the mean starting weight of the subjects in the atomoxetine group was 81 kg. The small number of subjects who started the study, and the even smaller number of those treated with atomoxetine who completed it (n = 4), affected the statistical power of the study and made the results far less reliable. Also, none of the study subjects was able to remain completely abstinent for the 21-day period, so even for those that could tolerate this drug, the success was not as beneficial as might be desired. The limited sample also prevented us from generalization and extrapolation of acquired results. Thus, our findings should be interpreted cautiously.

Fourthly, the duration of the study was 21 days, during which the effects of atomoxetine on nicotine withdrawal were examined. The duration of the study may not have been long enough to assess the effect of atomoxetine on nicotine withdrawal, especially, taking into account that the first 3 to 4 weeks of abstinence are considered to be an acute nicotine withdrawal period that represents the high-risk period for relapse [28]. Ideally, longer study periods would show the utility (or otherwise) of atomoxetine in achieving and maintaining abstinence. Thus, in future studies, a longer duration of atomoxetine administration may be required to determine any differences between the natural progression of nicotine withdrawal and the time required to observe the therapeutic effect of atomoxetine. Therefore, it should be appreciated that the length of treatment in the present study was experimental and was not sufficient enough to determine a definitive therapeutic effect of atomoxetine on smoking cessation.

Lastly, because the groups were small there were differences between them that could have affected the results. Thus, the atomoxetine and placebo group differed (even if not statistically significantly) in terms of gender, education, occupation, income, marital status, history of mental disorder, number with health concerns from smoking, use of medication for medical conditions, length they had been smoking, median length of attempts to quit smoking, median length of previous attempts to stop smoking, and helpfulness of previous medication for smoking cessation. While these differences may not have been statistically significant on an individual basis, in small groups of subjects such differences can conceivably have a significant cumulative effect that could impact the results. Consideration of the discussion of the results that follows should take all these study limitations into account.

## Conclusions

In conclusion, the data from the present study support suggestions that there may be some useful role for atomoxetine in the treatment of smoking cessation, particularly as the number of cigarettes per day decreased, and this was supported by a decrease in the serum cotinine levels. However, the size of the study and the limitations identified, mean that further work is required to determine this more definitively. One matter that will need to be address in future studies will involve both the dose used and the length of treatment.

## Competing interests

The authors declare that they have no competing interests.

## Authors' contributions

PHS conceived of the study, participated in its design and drafted the manuscript. RD participated in its design, carried out all measurements in the study, and helped to draft the manuscript. All authors read and approved the final manuscript, which in part formed the basis for a Masters Degree in Psychiatry awarded to RD.
